# The Association of Obesity and the Antiaging Humoral Factor Klotho in Middle-Aged and Older Adults

**DOI:** 10.1155/2022/7274858

**Published:** 2022-08-24

**Authors:** Carlos H. Orces

**Affiliations:** Laredo Medical Center, 1700 E Saunders St. Laredo, Laredo 78041, TX, USA

## Abstract

**Objectives:**

Previous studies have reported conflicting results regarding the relationship between obesity and the antiaging humoral factor klotho. Thus, the present study aimed to examine the association of anthropometric measurements, weight history change, and vitamin D status with serum klotho levels.

**Methods:**

The National Health and Nutrition Examination Survey data were used to compare sex-specific logarithm transformed serum klotho levels across standardized anthropometric measurements and weight change history in subjects aged 40–79 years. The baseline measured height, and self-reported weight were used to calculate the body mass index (BMI) at two-time intervals: age 25 and ten years before the measured BMI. Subjects were then categorized as never obese, obese to nonobese, nonobese to obese, and always obese.

**Results:**

Of 4,971 participants, the prevalence of general obesity was 41%, and abdominal obesity (waist circumference ≥88 cm) was present in 75% of women. Overall, lower serum klotho levels were seen in older adults, men, nonsmokers, alcohol users, and those with an estimated glomerular filtration rate (eGFR) < 60 ml/min. Multivariate models demonstrated that general and abdominal obesity in women was inversely associated with serum klotho levels. Moreover, women who developed obesity from age 25 and ten years before the baseline BMI had significantly lower mean klotho levels at 765.0 and 757.4 pg/ml compared with 820.5 pg/ml (*p* < 0.05) among those that were never obese, respectively. In contrast, serum klotho levels did not significantly differ among men, irrespective of their weight history. In a subanalysis, higher klotho levels were seen in participants with an adequate vitamin D status (≥50 nmol/L) than their overweight and obese counterparts (*p* < 0.05).

**Conclusions:**

Obesity among women was significantly and inversely associated with serum klotho levels. Similarly, women who developed obesity during their lifetime had consistently lower klotho levels than their never-obese counterparts.

## 1. Introduction

The klotho gene, expressed primarily in the distal tubule of the kidney, encodes a single-pass transmembrane protein and a soluble form resulting from proteolytic cleavage of the extracellular domain by membrane-anchored proteases [1]. The membrane klotho functions as an obligate coreceptor with fibroblast growth factor 23 (FGF23), a bone-derived hormone that inhibits phosphate reabsorption in the kidney and mediates regulation of vitamin D metabolism [2]. The soluble klotho is a humoral factor with several extra-renal pleiotropic effects [3].

Kuro-o et al. initially reported that a mutation of the mouse klotho gene produces a phenotype resembling premature aging [4]. Subsequently, several observational studies in older adults have demonstrated that lower serum klotho levels correlate with poor grip strength and decreased knee strength, prevalent cardiovascular disease, abdominal aortic calcification, and vascular dementia [5–9].

Although obesity is a significant public health problem associated with increased risk of cardiovascular disease, type 2 diabetes mellitus, cancer, mortality, and an inadequate vitamin D status, previous research has reported conflicting results regarding the association between obesity and serum klotho levels [10–14]. Indeed, sedentary middle-aged obese subjects had higher serum klotho levels than their normal body weight counterparts [12]. Likewise, increased serum klotho levels were reported among obese children and adolescents hospitalized with gastrointestinal symptoms [13]. In contrast, young Japanese women with restrictive-type anorexia nervosa or obesity had significantly lower serum klotho levels than those with a healthy body weight [14]. Recent studies also demonstrated that serum klotho levels were negatively correlated with waist circumference in young women with polycystic ovary syndrome and among community-dwelling adults with abdominal obesity [15–17]. Whether the obesity history is associated with lower klotho levels has not been previously explored. Thus, the present study aimed to examine the association of anthropometric measurements and weight change history with serum klotho levels in adults aged 40–79 years. A secondary objective was to determine the effect of the vitamin D status on serum klotho levels according to the body weight.

## 2. Material and Methods

### 2.1. Study Participants

The present study was based on participants in the National Health and Nutrition Examination Survey (NHANES) cycles 2013–2014 and 2015–2016. The NHANES is designed to continuously assess the health and nutritional status of the civilian noninstitutionalized U.S. population. The survey protocol was approved by the National Center for Health Statistics Research Ethics Review Board (study protocol^#^ 2011–17), and all participants gave informed consent. A detailed description of the methods and analytic guidelines are found at https://wwwn.cdc.gov/nchs/nhanes/analyticguidelines.aspx.

### 2.2. Covariates

The age, race/ethnicity, and education of participants were self-reported. Participants' smoking status was categorized as never, former, or current smoker. Alcohol use was considered if the subjects responded affirmatively to the question “Have you had at least 12 drinks of any type of alcoholic beverage in any single year? Physical activity was assessed by asking participants the number of days and minutes spent in recreational moderate- or vigorous-intensity aerobic physical activity in a typical week. Those who met the Physical Activity Guidelines for Americans were considered to be physically active [18]. Diabetes mellitus was considered to be prevalent if participants reported a physician diagnosis of diabetes or had a hemoglobin A1c ≥ 6.5% [19]. The DxC800 modular chemistry side was used to analyze serum creatinine (mg/dl). Subsequently, the estimated glomerular filtration rate (eGFR) was calculated according to the modification of diet in renal disease formula [20]. Serum 25 and hydroxyvitamin D (25 (OH)D) concentrations (nmol/l) were measured by using a standardized liquid chromatography-tandem mass spectrometry method. 25 (OH) D inadequacy (<50 nmol/L) was defined according to the Institute of Medicine 25 (OH) D cutoff points relative to bone health [21].

### 2.3. Anthropometric Measurements

In the mobile examination center, standing height was measured using a stadiometer with a fixed vertical board and an adjustable headpiece. Subjects wore a standard gown and their weight in kilograms was recorded using a digital weight scale. The body mass index (BMI) was calculated as weight in kilograms divided by height in meters squared and rounded to one decimal place. BMI was categorized as normal weight (BMI, 18.5 to < 25 kg/m^2^), overweight (BMI, 25 to < 30 kg/m^2^), and obesity (BMI ≥30 kg/m^2^). Because a small number of participants (*n* = 48) were defined as underweight (BMI <18.5 kg/m^2^), they were grouped into the normal weight category. The waist circumference (cm) was measured with participants standing on the right side. Technicians then marked the right iliac crest with a cosmetic pencil and extended the measuring tape around the waist. Waist circumference cut-points ≥102 cm in men and ≥88 cm in women were used to define abdominal obesity [22].

### 2.4. Weight Change History

Trained interviewers assessed participants' weight history by asking, “How much did you weigh 10 years ago? and “How much do you weigh at age 25? If you do not know your exact weight, please make your best guess.” Self-reported weight history in pounds was converted to kilograms. The measured height at baseline was used to calculate the BMI at age 25 and 10 years prior. As previously described, weight change history was calculated for two-time intervals: BMI at age 25 and baseline BMI and BMI 10 years before the baseline BMI. The weight change pattern was then categorized as never obese, obese to nonobese, nonobese to obese, and always obese [23].

### 2.5. Serum Soluble Klotho

Participants aged 40–79 years in the NHANES 2013–2014 and 2015–2016 cycles gave consent for their frozen blood samples (surplus) stored at −80°C to be used in future research. Subsequently, soluble klotho levels (pg/ml) were analyzed with a commercially available ELISA kit produced by IBL International, Japan, during the period 2019-2020. All analyses were performed at the University of Washington research laboratories. A completed description of the laboratory methodology can be found at https://wwwn.cdc.gov/Nchs/Nhanes/2013-2014/SSKL_H.htm.

### 2.6. Statistical Analysis

The Shapiro–Wilk test was used to assess data normality. Because serum klotho levels had a right-skewed distribution, a natural logarithm (ln) transformation was performed to achieve a normal distribution. For practical use, the results of the statistical analysis were back-transformed to the original soluble klotho levels in pg/ml. The mean klotho levels were compared with the *t*-test and ANOVA according to the characteristics of participants. Moreover, sex-specific general linear models adjusted for age, race/ethnicity, education, smoking status, alcohol use, physical activity, diabetes, 25 (OH)D levels, and eGFR were assembled to examine serum klotho levels across BMI categories, abdominal obesity status, and weight change pattern. In a subanalysis, the effect of the vitamin D status on klotho levels was analyzed by BMI categories. SPSS Complex Sample software V17 was used to account for the NHANES complex survey design. Of 6,853 participants aged 40–79 years, those with missing data on eGFR, serum klotho, and anthropometric measurements were excluded (*n* = 1,882).

## 3. Results

A total of 4,971 participants with a mean age of 57.4 (SD 10.6) years comprised the study sample. As shown in Table 1, general obesity was prevalent in 41.8% of participants. Notably, about 33% and 16% of participants developed obesity from age 25 and 10 years before the baseline measured BMI, respectively. Moreover, abdominal obesity was present in 75.7% of women compared with 55.8% of men. In general, significantly lower serum klotho levels were seen in adults aged 60 years and older, men, overweight and obese subjects, women with abdominal obesity, nonsmokers, alcohol users, and those with an eGFR <60 ml/min.

**Figure 1:**
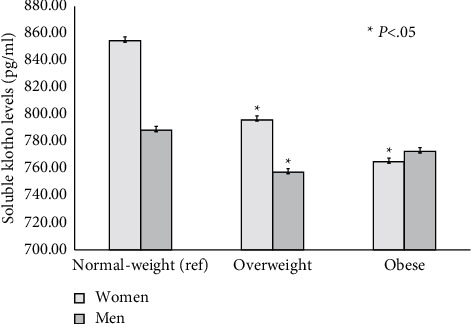
Sex-specific BMI categories and serum soluble klotho levels.

**Figure 2:**
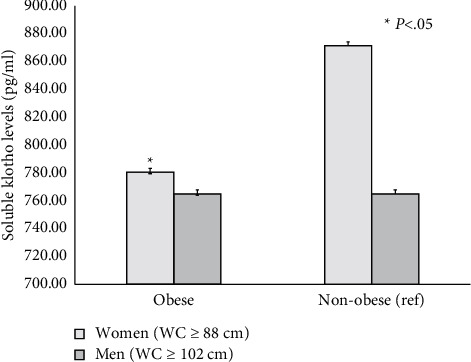
Sex-specific abdominal obesity prevalence and serum soluble klotho levels.

**Figure 3:**
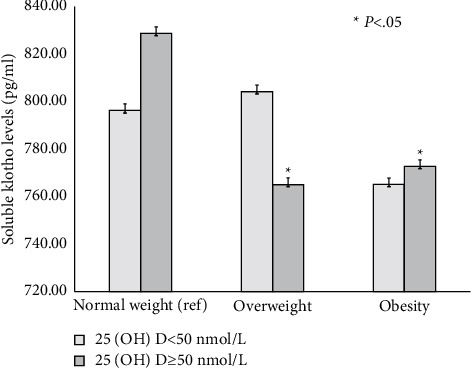
Soluble Klotho levels stratified according to the vitamin D status and BMI categories.

As shown in Figure 1, serum soluble klotho levels in women were significantly and inversely associated with BMI categories. Moreover, women defined as having abdominal obesity had lower klotho levels compared with their nonabdominally obese counterparts (*p* < 0.05). In contrast, similar klotho levels were seen in men, irrespective of their abdominal obese status (Figure 2). As shown in Figure 3, among participants with an adequate vitamin D status, serum klotho levels were higher in those with a healthy body weight compared with their overweight and obese counterparts. However, serum klotho levels did not significantly differ across BMI categories in subjects with vitamin D inadequacy.

Table 2 shows sex-specific weight change patterns and serum klotho levels. After adjustment for potential confounders, women categorized as always obese or who developed obesity ten years before the baseline BMI had on average 40 and 63.1 pg/ml lower serum klotho levels compared with their never-obese counterparts, respectively. Similarly, non-obese women at the age of 25 who developed obesity later in life had 55.5 pg/ml lower klotho levels than those that were never obese. In contrast, comparable klotho levels were seen in men, irrespective of their weight change pattern.

## 4. Discussion

This cross-sectional study demonstrates that general and abdominal obesity in middle-aged and older women were significantly associated with low serum klotho levels. Notably, even after adjustment for potential confounders, women who developed obesity from the age of 25 and ten years before the baseline BMI had consistently lower klotho levels than their never-obese counterparts. In contrast, serum klotho levels did not significantly differ in men, irrespective of their obesity history.

A few earlier studies reported conflicting results regarding the relationship between BMI and klotho levels. Amaro-Gahete et al. demonstrated a significant age- and sex-adjusted positive association between BMI and serum klotho levels among middle-aged sedentary subjects, which disappeared after adjustment for lean mass. In that latter study, the authors concluded that skeletal muscle tissue might play a significant role in klotho metabolism [10]. Moreover, among older participants in the Invecchiare in the Chianti (InCHIANTI) study, BMI was comparable across serum klotho tertile levels [4]. In contrast, young Japanese women with a mean BMI of 35.7 kg/m^2^ had significantly lower serum klotho levels than their healthy body weight counterparts, which is consistent with the study findings [14]. Likewise, serum klotho levels were negatively correlated with waist circumference and BMI among women aged 19–33 years with polycystic ovary syndrome [15]. A recent large analysis of the NHANES also demonstrated significantly lower serum klotho levels among participants with abdominal obesity than those without [16]. The present results extend those from previous research by reporting that prevalent general and abdominal obesity were significantly associated with lower klotho levels only in women. Although prior research has demonstrated higher klotho levels in women and younger age groups; sex differences regarding serum klotho levels in obese subjects might be explained by a higher percentage of fat mass in women than that in men and the effect of estrogen promoting adipocyte precursor proliferation in both visceral and gluteofemoral regions [24]. The physiological effects of sex steroid hormones on klotho levels have not been completely elucidated. However, Dote–Montero et al. reported that testosterone concentrations were positively correlated with serum klotho levels in sedentary middle-aged men and women with a mean BMI of 26.7 kg/m^2^ [25]. Of relevance, after adjusting for age, the strength of association disappeared in men and remained unchanged in women.

Observational studies have consistently demonstrated lower 25 (OH)D levels in adults with obesity, which are attributed to limited sun exposure, inadequate use of vitamin D supplements, or simply the dilution of ingested or synthesized vitamin D [26, 27]. Notably, Wojcik et al. reported that among obese adolescents with normal renal function, subjects with 25 (OH)D levels <50 nmol/L had marginally lower FGF-23 concentrations than those with adequate 25 (OH)D levels. The authors suggested that low vitamin D levels may cause a slight increase in PTH levels which may inhibit renal phosphate reabsorption and indirectly FGF-23 secretion [28]. Of interest, higher serum klotho levels were seen in subjects with normal weight compared with their overweight and obese counterparts with adequate vitamin D status. In contrast, serum klotho levels did not significantly differ across BMI categories in participants with vitamin D inadequacy. A previous study demonstrated that 25 (OH)D levels were on average 5.1 nmol/L lower among subjects aged 40 years and older who gained weight ≥5% in the previous ten years than those who maintained a stable weight, even after adjustment for vitamin D supplements [27]. Thus, it is feasible that vitamin D may potentially mediate the relationship between obesity and serum klotho levels.

The present study has several limitations that should be mentioned. First, because of its cross-sectional design, the association between obesity and serum klotho levels do not necessarily infer causation. Second, the study findings may be only generalized to subjects aged 40–79 years. Third, BMI at age 25 and ten years before baseline was calculated according to participants' self-reported weight, which may be a source of recall bias. Finally, data on body composition were not available for this analysis. Despite these limitations, the large sample size representative of community-dwelling middle-aged and older adults is the main strength of the present study.

In conclusion, general and abdominal obesity were inversely associated with serum klotho levels in women. Similarly, women who developed obesity during their lifetime had consistently lower klotho levels than their never-obese counterparts. Further studies are indicated to elucidate the effect of adipose tissue on serum klotho.

## Figures and Tables

**Table 1:** Characteristics of participants and serum soluble klotho levels.

	%	Klotho levels (pg/ml)	*P* value
Age (years)			<0.0001
40–59	55.4	804.3	
≥60	44.6	757.4	

Sex			<0.05
Men	47.9	765.0	
Women	52.1	796.3	

Race/ethnicity			<0.05
Mexican-American	6.9	804.3	
Other Hispanic	4.9	828.8	
NHW	72.1	772.8	
NHB	8.6	820.6	
Multiracial	7.5	812.4	

Education			<0.001
≤11th grade	12.9	796.3	
High school graduate	20.8	742.5	
Some college	32.2	772.8	
College graduate	34.1	804.3	

BMI (kg/m2)			<0.0001
Normal weight	23.8	820.6	
Overweight	34.4	765.1	
Obese	41.8	772.8	

Abdominal obesity in men			0.939
Yes	55.8	765.0	
No	44.2	765.0	

Abdominal obesity in women			<0.0001
Yes	75.7	780.5	
No	24.3	862.6	

BMI 25 years to BMI baseline			<0.05
Never obese	56.8	788.3	
Obese to nonobese	1.4	788.3	
Nonobese to obese	33.6	765.0	
Always obese	8.2	804.3	

BMI 10 years prior to BMI baseline			<0.05
Never obese	52.3	796.3	
Obese to nonobese	5.9	765.0	
Nonobese to obese	16.9	765.0	
Always obese	24.9	780.5	

Smoking status			<0.0001
Never	52.9	804.3	
Former	29.8	772.7	
Current	17.3	742.4	

Alcohol use			<0.0001
Yes	78.3	772.7	
No	21.7	812.4	

Physical activity			0.499
Yes	33.2	834.4	
No	66.8	826.1	

Diabetes			0.597
Yes	16.6	828.9	
No	83.4	828.7	

eGFR (ml/min)			<0.0001
<60	10.8	736.7	
≥60	89.2	839.8	

25(OH)D (nmol/L)			0.835
<50	17.9	829.6	
≥50	82.1	828.5	

NHW : Non-Hispanic white; NHB: mon-Hispanic black; BMI: body mass index, eGFR: estimated glomerular filtration rate.

**Table 2:** Weight change pattern and serum soluble klotho levels (pg/ml) in men and women aged 40–79 years.

	Never obese (ref)	Obese to nonobese	Nonobese to obese	Always obese
Men				
BMI 10 years prior to baseline BMI				
Model 1	765.0	742.4	757.4	780.5
Model 2	765.0	742.4	765.0	780.5
Model 3	765.0	735.0	765.0	772.7
BMI age 25 years to baseline BMI				
Model 1	765.0	749.9	765.0	796.3
Model 2	765.0	757.4	765.0	788.3
Model 3	765.0	765.0	765.0	772.7

Women				
BMI 10 years prior to baseline BMI				
Model 1	820.5	812.4	765.0^*∗*^	788.3^*∗*^
Model 2	820.5	820.5	757.4^*∗*^	780.5^*∗*^
Model 3	820.5	828.8	757.4^*∗*^	780.5^*∗*^
BMI age 25 years to baseline BMI				
Model 1	820.5	871.3	772.7^*∗*^	796.3
Model 2	820.5	897.8	765.0^*∗*^	796.3
Model 3	820.5	888.9	765.0^*∗*^	796.3

^*∗*^ *P* < 0.05 compared with the reference category never obese. Model 1: adjusted for age- and gender, model 2: adjusted for model 1 and race/ethnicity, education, smoking, alcohol use, and physical activity, and model 3: adjusted for model 2 and diabetes, eGFR, and 25 (OH)D levels.

## Data Availability

The data used to conduct the present study may be available upon request.

## References

[1] Kuro-o M. (2011). Klotho and the aging process. *Korean Journal of Internal Medicine (Korean Edition)*.

[2] Latic N., Erben R. G. (2021). FGF23 and vitamin D metabolism. *JBMR Plus*.

[3] Hu M. C., Kuro-o M., Moe O. W. (2013). Renal and extrarenal actions of Klotho. *Seminars in Nephrology*.

[4] Kuro-o M., Matsumura Y., Aizawa H. (1997). Mutation of the mouse klotho gene leads to a syndrome resembling ageing. *Nature*.

[5] Semba R. D., Cappola A. R., Sun K. (2012). Relationship of low plasma klotho with poor grip strength in older community-dwelling adults: the InCHIANTI study. *European Journal of Applied Physiology*.

[6] Semba R. D., Ferrucci L., Sun K. (2016). Low plasma klotho concentrations and decline of knee strength in older adults. *The Journals of Gerontology Series A: Biological Sciences and Medical Sciences*.

[7] Orces C. H. (2022). The association between serum soluble klotho levels and abdominal aorta calcification in older adults. *Aging Clinical and Experimental Research*.

[8] Semba R. D., Cappola A. R., Sun K. (2011). Plasma klotho and cardiovascular disease in adults. *Journal of the American Geriatrics Society*.

[9] Brombo G., Bonetti F., Ortolani B. (2018). Lower plasma klotho concentrations are associated with vascular dementia but not late-onset Alzheimer’s disease. *Gerontology*.

[10] de Mutsert R., Sun Q., Willett W. C., Hu F. B., van Dam R. M. (2014). Overweight in early adulthood, adult weight change, and risk of type 2 diabetes, cardiovascular diseases, and certain cancers in men: a cohort study. *American Journal of Epidemiology*.

[11] Samuel L., Borrell L. N. (2013). The effect of body mass index on optimal vitamin D status in U.S. adults: the National Health and Nutrition Examination Survey 2001-2006. *Annals of Epidemiology*.

[12] Amaro-Gahete F. J., De-la-O A., Jurado-Fasoli L. (2019). Body composition and S-Klotho plasma levels in middle-aged Adults: a cross-sectional study. *Rejuvenation Research*.

[13] Socha-Banasiak A., Michalak A., Pacześ K. (2020). Klotho and fibroblast growth factors 19 and 21 serum concentrations in children and adolescents with normal body weight and obesity and their associations with metabolic parameters. *BMC Pediatrics*.

[14] Amitani M., Asakawa A., Amitani H. (2013). Plasma klotho levels decrease in both anorexia nervosa and obesity. *Nutrition*.

[15] Biyik I., Erten O., Isiklar O. O. (2021). Comparison of serum human Klotho levels and thiol/disulfide homeostasis in women with polycystic ovary syndrome and in healthy women. *Taiwanese Journal of Obstetrics & Gynecology*.

[16] Cheng Y. W., Hung C. C., Fang W. H., Chen W. L. (2022). Association between soluble *α*-Klotho protein and metabolic syndrome in the adult population. *Biomolecules*.

[17] Orces C. H. (2022). The association between metabolic syndrome and the anti-aging humoral factor klotho in middle-aged and older adults. *Diabetes & Metabolic Syndrome: Clinical Research Reviews*.

[18] (2022). https://health.gov/our-work/nutrition-physical-activity/physical-activity-guidelines/current-guidelines.

[19] American Diabetes Association (2010). Diagnosis and classification of diabetes mellitus. *Diabetes Care*.

[20] National Kidney Foundation (2002). K/DOQI clinical practice guidelines for chronic kidney disease: evaluation, classification, and stratification. *American Journal of Kidney Diseases*.

[21] Institute of Medicine (US) (2011). Committee to Review dietary reference intakes for vitamin D and calcium. *Dietary Reference Intakes for Calcium and Vitamin D*.

[22] Alberti K. G., Eckel R. H., Grundy S. M. (2009). Harmonizing the metabolic syndrome: a joint interim statement of the international diabetes federation task force on epidemiology and prevention. *Circulation*.

[23] Chen C., Ye Y., Zhang Y., Pan X. F., Pan A. (2019). Weight change across adulthood in relation to all cause and cause specific mortality: prospective cohort study. *BMJ*.

[24] Gavin K. M., Bessesen D. H. (2020). Sex differences in adipose tissue function. *Endocrinology and Metabolism Clinics of North America*.

[25] Dote-Montero M., Amaro-Gahete F. J., De-la-O A., Jurado-Fasoli L., Gutierrez A., Castillo M. J. (2019). Study of the association of DHEAS, testosterone and cortisol with S-Klotho plasma levels in healthy sedentary middle-aged adults. *Experimental Gerontology*.

[26] Palacios C., Gil K., Pérez C. M., Joshipura K. (2012). Determinants of vitamin D status among overweight and obese Puerto Rican adults. *Annals of Nutrition & Metabolism*.

[27] Orces C. H. (2020). The relationship between weight change history and 25(OH)D concentrations in adults. *Nutricion Hospitalaria*.

[28] Wojcik M., Janus D., Kalicka-Kasperczyk A., Sztefko K., Starzyk J. B. (2017). The potential impact of the hypovitaminosis D on metabolic complications in obese adolescents—preliminary results. *Annals of Agricultural and Environmental Medicine*.

